# Identification of the *bla*_OXA-23_ Gene in the First Mucoid XDR *Acinetobacter baumannii* Isolated from a Patient with Cystic Fibrosis

**DOI:** 10.3390/jcm12206582

**Published:** 2023-10-18

**Authors:** Martina Rossitto, Gianluca Vrenna, Vanessa Tuccio Guarna Assanti, Nour Essa, Maria Luisa De Santis, Annarita Granaglia, Vanessa Fini, Valentino Costabile, Manuela Onori, Luca Cristiani, Alessandra Boni, Renato Cutrera, Carlo Federico Perno, Paola Bernaschi

**Affiliations:** 1 Multimodal Laboratory Medicine, Bambino Gesù Children’s Hospital, IRCCS, 00165 Rome, Italy; 2 Major School in Microbiology and Virology, University Campus Bio-Medico, 00128 Rome, Italy; 3 Microbiology and Diagnostic Immunology Unit, Bambino Gesù Children’s Hospital, IRCCS, 00165 Rome, Italy; vanessa.tuccio@opbg.net (V.T.G.A.); nour.essa@opbg.net (N.E.); marialuisa.desantis@opbg.net (M.L.D.S.); annarita.granaglia@opbg.net (A.G.); vanessa.fini@opbg.net (V.F.); valentino.costabile@opbg.net (V.C.); manuela.onori@opbg.net (M.O.); carlofederico.perno@opbg.net (C.F.P.); paola.bernaschi@opbg.net (P.B.); 4 Department of Molecular Medicine, Sapienza University of Rome, 00185 Rome, Italy; 5 Pneumology and Cystic Fibrosis Unit, Bambino Gesù Children Hospital, IRCCS, 00165 Rome, Italy; luca.cristiani@opbg.net (L.C.); alessandra.boni@opbg.net (A.B.); renato.cutrera@opbg.net (R.C.)

**Keywords:** *Acinetobacter baumannii*, beta-lactamase OXA-23, cystic fibrosis, mucoid, multidrug resistance

## Abstract

*Acinetobacter baumannii* is one of the pathogens most involved in health care-associated infections in recent decades. Known for its ability to accumulate several antimicrobial resistance mechanisms, it possesses the oxacillinase *bla*_oxa-23_, a carbapenemase now endemic in Italy. *Acinetobacter* species are not frequently observed in patients with cystic fibrosis, and multidrug-resistant *A. baumannii* is a rare event in these patients. Non-mucoid *A. baumannii* carrying the *bla*_oxa-23_ gene has been sporadically detected. Here, we describe the methods used to detect *bla*_oxa-23_ in the first established case of pulmonary infection via a mucoid strain of *A. baumannii* producing carbapenemase in a 24-year-old cystic fibrosis patient admitted to Bambino Gesù Children’s Hospital in Rome, Italy. This strain, which exhibited an extensively drug-resistant antibiotype, also showed a great ability to further increase its resistance in a short time.

## 1. Introduction

*Acinetobacter baumannii* (*A. baumannii*) is one of the most important opportunistic multidrug-resistant (MDR) pathogens. With its remarkable ability to rapidly unleash new pathogenic mechanisms, such as horizontally acquired resistance determinants, and survive in the environment for prolonged periods, *A. baumannii* has established a niche in the hospital environment [1]. Acknowledging its potential threat to human health, in 2017, the World Health Organization included carbapenem-resistant *A. baumannii* among the critical microorganisms for which new antibiotics are urgently needed [2]. Growing evidence of extensively drug-resistant (XDR) and pandrug-resistant (PDR) isolates of *A. baumannii* is indeed accumulating in different countries [3]. Several severe infections sustained by resistant *A. baumannii* have been observed in southern European countries (Greece, Italy and Spain), particularly in patients with severe comorbidities in intensive care units (ICUs) [1]. To date, among the *A. baumannii* clones distributed worldwide, the international clone ST2 is the most dominant type [4,5]. Recently, many isolates of *A. baumannii* have been identified as mucoid-type [6], and in a recent study, most of the mucoid strains belonged to the ST2 clone of *A. baumannii* [7]. In addition, several studies have demonstrated the prevalence of isolates belonging to this clone that mainly harbor the *bla*_oxa-23_ oxacillinase [8]. Oxacillinases (OXAs), class D carbapenemases according to Ambler’s scheme, are the main resistance mechanism to carbapenems in *A. baumannii.* Among the oxacillinases possessed and acquired by *A. baumannii*, the variants from the OXA-23-like family have spread worldwide, becoming the predominant carbapenamases in several areas [9].

Although *A. baumannii* has a recognized role as a health pathogen, it is not considered a classical pathogen in cystic fibrosis (CF), a genetic disease that causes abnormal thickening of lung mucus that becomes a breeding ground for bacterial colonization [10]. Indeed, current infections and chronic colonization with *Pseudomonas aeruginosa, Staphylococcus aureus, Haemophilus influenzae, Stenotrophomonas maltophila, Achromobacter xylosoxidans* and the *Burkholderia cepacia* complex are commonly observed in CF patients [11]. On the contrary, *A. baumannii* has rarely been described as colonizing the airways of CF patients. Apart from the occasional finding of post-transplant infection caused by a donor lung infected with *A. baumannii* producing OXA-23 carbapenemase [12], only one study describes the presence of nonmucoid MDR *A. baumannii* harboring *bla*_oxa-23_ in a small set of Brazilian CF patients [10].

Here, we describe for the first time the isolation of a mucoid strain of *A. baumannii* producing OXA-23, a phenotype never occurred in a patient with CF, and the *bla*_oxa-23_ identification process.

## 2. Case Description

We present here the case of a 24 year-old Caucasian female with a delayed CF diagnosis in adulthood due to follow-up interruption at the regional CF center after an initial positive newborn screening and an inadequate sweat chloride sample at 3 months. The formal referral to our CF center was suggested after a prolonged history of persistent respiratory symptoms, chronic sputum, recurrent low respiratory tract infections treated with oral antibiotics, a computer-tomography scan with evidence of diffuse bronchiectasis and a spontaneous tension pneumothorax at 22 years of age, treated with a right upper lobe segmentectomy and chest tube drainage with a 10-day ICU stay at a local hospital. A bronchoalveolar lavage was also performed during that period, which resulted in the isolation of *Candida albicans*, mucoid *P. aeruginosa* and *Klebsiella pneumoniae*.

During initial evaluation in our center, a CF diagnosis was made in light of a positive sweat chloride test (105 mEq/L) and the detection of two CF-causing CFTR mutations (homozygous dele14b-17b). In addition, the patient was underweight (BMI 16 kg/m^2^), with exocrine pancreas insufficiency (Fecal Elastase-1 < 50 µg/g). The initial assessment also demonstrated a Forced Expiratory Volume in the first second (FEV1) of 31%, Vitamin D insufficiency (19.6 ng/mL) and elevated glycated hemoglobin (43 mmol/mol). Treatment of the respiratory exacerbation was promptly initiated on the basis of a previous sputum culture positive for *P. aeruginosa*, with a 14-day intravenous antibiotic course of meropenem 2 g BID and tobramycin 10 mg/kg once daily, with a good clinical response. At hospital discharge, continuous alternating inhaled antibiotic therapy with tobramycin and colistin was also prescribed as a standard of care in the maintenance treatment of the chronic *P. aeruginosa* infection.

## 3. Microbiological Investigation

### 3.1. Isolates and Antimicrobial Susceptibility Testing

**Figure 1:**
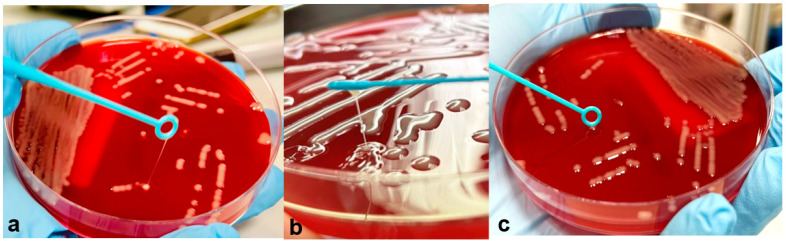
String test of the *A. baumannii* hypermucoid strain (HM) and a detail of its string (**a**,**b**); string test of the *A. baumannii* low-mucoid strain (LM) (**c**).

During the patient’s stay at our hospital, standard sputum culture was performed according to specific guidelines for CF patient respiratory sample investigation [13]. Accordingly, the patient’s sputum was liquefied with the appropriate amount of sputum liquefying solution (Copan, Brescia, Italy). The following culture media were inoculated for the semiquantitative count of colony forming units (CFU): MacConkey agar, Mannitol salt 2 agar, Burkholderia Cepacia selective agar, Columbia agar + 5% sheep blood, Haemophilus Chocolate 2 agar, Columbia CNA agar + 5% sheep blood, Sabouraud Gentamicin Chloramphenicol 2 agar (bioMérieux, Marcy l’Etoile, France), Pseudosel agar (Cetrimide agar) (Becton Dickinson GmbH, Heidelberg, Germany), Scedosporium selective agar (Liofilchem, Roseto degli Abruzzi, Italy). Colonies growing on agar plates were considered putative pathogens based on their morphologic characteristics and fermenting/non-fermenting ability on the respective culture media. They were subcultured and subsequently identified via Matrix-Assisted Laser Desorption Ionization Time-of-Flight Mass Spectrometry (MALDI-TOF MS; Bruker Daltonics, Bremen, Germany). Hence, mucoid *P. aeruginosa, A. xylosoxidans, C. albicans* and two strains of *A. baumannii* with different colony morphologies, one being mucoid and the other with smooth rounded margins, were recovered from patient sputum culture. Both *A. baumannii* isolates were then cultured overnight on blood agar plates at 37 °C, and bacterial colonies were then stretched with an inoculation loop for the string test to assess their mucoidity [6]. According to Gong and colleagues [6], the two strains were classified as hypermucoid (HM) and low mucoid (LM), with viscous strings of 150 mm and 0 mm in length, respectively (Figure 1).

Antibiograms for both strains were performed via the broth microdilution method using the Sensititre Gram Negative DMKGN plate (ThermoFisher Scientific, Waltham, MA, USA) and ComASP^®^ cefiderocol (Liofilchem, Roseto degli Abruzzi, Italy), and were interpreted according to clinical breakpoints based on the European Committee on Antimicrobial Susceptibility Testing (EUCAST) tables (version 13.0) [14]. Both the LM and HM *A. baumannii* strains showed XDR profiles for both isolates, with sensitivity exclusively to colistin and low MICs to tigecycline and cefiderocol (Table 1).

### 3.2. Carbapenemase Detection

Therefore, the following tests were performed: (i) NG-Test Carba 5 (NG Biotech, Guipry, France) to rapidly detect the five main carbapenemases (i.e., KPC, OXA-48-like, NDM, VIM and IMP), (ii) modified Hodge test, using *Escherichia coli* ATCC 25922 as indicator organism, *A. baumannii* ATCC 17978 as negative control and a clinical strain of *Klebsiella pneumoniae* KPC as positive control, (iii) real-time PCR Xpert^®^ Carba (Cepheid, Sunnyvale, CA, USA) for rapid detection and differentiation of five genes (i.e., *bla*_KPC_, *bla*_VIM_, *bla*_OXA-48_, *bla*_IMP-1_ and *bla*_NDM_). The NG-Test Carba 5 gave an uninterpretable result. The modified Hodge test revealed not carbapenemase production for the HM and LM *A. baumannii* strains. The PCR Xpert^®^ Carba came back negative.

Hence, we performed a homemade PCR for the *bla*_oxa-23_ gene as previously described with some modifications [9]. Namely, bacterial DNA was extracted using the automatic extractor EZ1 (Qiagen BioRobot EZ1, Qiagen, Hilden, Germany), with the extraction kit (EZ1&2 DNA tissue kit, Qiagen), following the manufacturer’s instructions and setting the elution volume at 50 µL. The extracted DNA was used for the homemade PCR. In particular, 2 µL of DNA template were added to 23 µL of reaction mixture, which contained 2.5 µL of PCR buffer 10× (10 mM Tris HCl, 25 mM KCl), 1 mM of MgCl2, 0.5 µL of each primer, 100 µM of each deoxynucleotide triphosphate (dATP, dGTP, dCTP and dTTP) and 1.5 U of Taq polymerase (Thermo Fisher Scientific, Waltham, MA, USA). As in the work of Corrêa and colleagues [9], we used primers with the sequences 5′-GATCGGATTGGAGAACCAGA-3′ and 5′-ATTTCTGACCGCATTTCCAT-3′. The reaction was carried as follows: a first step of initial denaturation at 94 °C for 5 min; followed by 30 cycles of 94 °C for 1 min, 54 °C for 1 min and 72 °C for 1 min; and the final extension step of 72 °C for 5 min.

**Figure 2:**
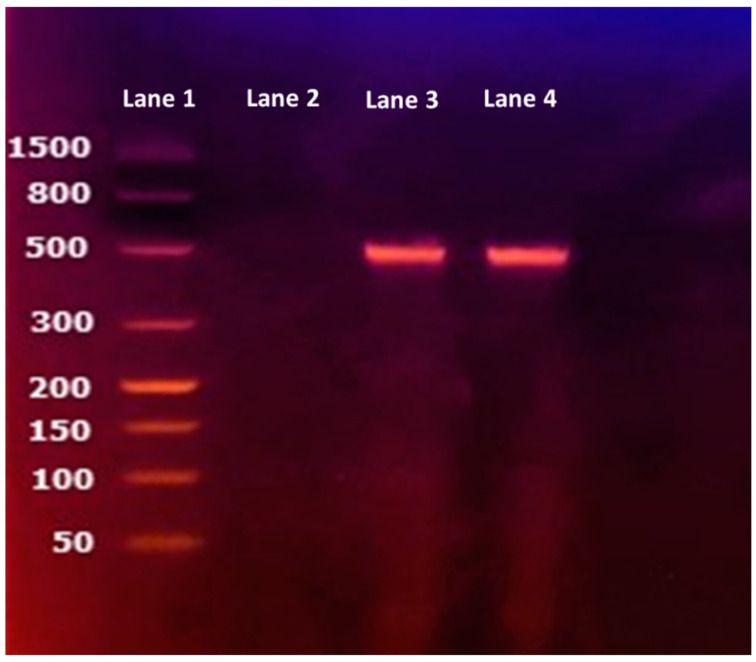
Lane 1: Ladder, Lane 2: negative control (PCR reaction mixture), Lane 3: HM *A. baumannii*, Lane 4: LM *A. baumannii*.

Amplification products were resolved and visualized directly on a closed ready-to-use 2.2% agarose gel-cassette system (FlashGel—Lonza, Basilea, Switzerland) using the 50–1500 bp FlashGel DNA marker (Lonza, Basilea, Switzerland). Fragments obtained from the two strains showed the presence of a single band of about 500 bp concordant with the size of the *bla*_oxa-23_ amplicon (Figure 2).

Subsequently, RESIST ACINETO immunochromatographic assay (Coris BioConcept, Gembloux, Belgium) for the detection of OXA-23, OXA-40/58 and NDM carbapenemases was acquired, in light of these results and to speed up *bla*_oxa-23_ identification, and tested. Consistent with the PCR result, it turned out to be positive for OXA-23.

### 3.3. Molecular Typing

Whole genome sequencing (WGS) through the Illumina platform (Illumina, San Diego, CA, USA) was also performed, in order to identify, among other features, the Sequence Type (ST) of both strains. Bacterial DNA extraction was performed as described for the homemade PCR for the *bla*_oxa-23_ gene. Molecular typing of the isolates was conducted to determine the genetic clonality relationship among the clinical isolates by using the seven housekeeping genes (*cpn60, fusA, gltA, pyrG, recA, rplB* and *rpoB*) according to the ST_Pasteur scheme. Multilocus sequence typing (MLST) analysis showed that both isolates belong to the same ST2 clone.

## 4. Discussion

Although *A. baumannii* is a widespread pathogen in health care facilities, it is not a commonly described pathogen in cystic fibrosis. With the exception of sporadic findings of nonmucoid MDR *A. baumannii* producing OXA-23 and belonging to the ST2 clone, pulmonary colonization in CF from a mucoid strain of this clone had never been described so far. The 24-year-old CF patient returned to our center after undergoing segmentectomy for right spontaneous hypertensive pneumothorax with a new infection with two different phenotypes of XDR *A. baumannii* belonging to ST2 and producing OXA-23 carbapenamase. Being the worldwide-distributed MDR *A. baumannii* clone ST2, a known culprit of nosocomial infections, we hypothesized that this infection was caused by the patient’s admission to the ICU following the lung segmentectomy. As recently noted, patients with CF may develop numerous conditions requiring ICU admission, including pneumothorax [15], and the growing number of adults with CF is significantly increasing ICU use [16]. In the paucity of data on the optimal ICU care of patients with CF, our observation draws attention to complications associated with ICU stay, such as microbial overinfection with nosocomial pathogens, which should be considered.

Previous researchers worldwide have reported increased resistance of the ST2 clone of *A. baumannii* to antibiotics, probably related to the presence of more resistance genes and mobilizable elements [1,4,8]. Consistently, the *A. baumannii* we isolated, belonging to ST2, showed multiresistance profiles and possessed the resistance enzyme OXA-23. 

The HM *A. baumannii* strain appeared to have the same characteristics as the strains tested in a recent study, in which mucoid *A. baumannii* isolates were resistant to most antibiotics except tigecycline and colistin (cefiderocol was not tested) and belonged to type ST2 [7]. As suggested by Shan and colleagues [7], mucoid *A. baumannii* could evolve from nonmucoid multidrug-resistant strains under adverse growth conditions, and this could be the reason for the coexistence of two strains (HM and LM) with different mucoidities. In fact, the patient’s low FEV1 and preexisting colonization by a mucoid phenotype of *P. aeruginosa* suggest the existence of a stressful lung environment with hypoxic mucus zones, which may have driven the bacteria to evolve toward a mucoid phenotype [17].

It is interesting to note that after five months, and during the writing of this paper, the patient returned for follow-up and a new sputum culture examination was performed. In this new sample, the LM phenotype of *A. baumannii* is no longer detectable, whereas the mucoid phenotype shows heteroresistance to colistin and cefiderocol. In order to explain the LM phenotype disappearance, we can speculate that, lacking the protection of a thick capsule, this strain was affected by intravenous and inhaled antibiotic therapies. Alternatively and more intriguingly, we can hypothesize that the LM phenotype may have acquired the ability to hyperproduce the capsule under the antibiotic selective pressure, thus becoming phenotypically indistinguishable from the HM. WGS analysis of the HM strains isolated in the second sample will hopefully allow us to discriminate between the two hypotheses.

Genomic analysis will also determine if the strain resistant to colistin in the second sample is an evolution of the previously susceptible strain. Resistance to colistin seems to easily appear from colistin-susceptible heteroresistant *A. baumannii* during treatment [18]; in our case, this phenomenon could be traced to the use of colistin in aerosol therapy, as prescribed to the patient. If this rapid evolution is confirmed, our observation might raise some concerns about the use of colistin, a standard of care in the maintenance treatment of chronic *P. aeruginosa* infection, when coinfections with microorganisms with such resistance profiles are present. Although there is no evidence in the literature that *A. baumannii* should be treated in CF [19], a microorganism with a similar resistance profile in a patient with severe ventilatory deficit and pulmonary parenchymal impairment requires careful antimicrobial management. In contrast, cefiderocol was never administered to the patient, but the strain that developed resistance to colistin also appears to be resistant to cefiderocol. This latter troubling resistance, lately increasingly reported, is generally related to the administration of the drug [20], contrary to what happened in the case presented here. Cefiderocol resistance may result from several mechanisms acting in concert (i.e., coexpression of different β-lactamases, siderophore receptor and porin expression mutations, efflux pump overexpression and target (PBP-3) modification); furthermore, isolates exhibiting carbapenem and extended-spectrum cephalosporin resistance present high prevalence of heteroresistance [14]. Another possible explanation could be the existence of a hypermutator phenotype within the *A. baumannii* population that may have been favorably selected during exposure to antibiotics [21].

## 5. Conclusions

*A. baumannii* is a pathogen rarely observed in CF and it is really uncommon among CF patients treated in our hospital. Therefore, and consistently with the epidemiology of our CF population, we did not have specific kits for *A. baumannii* carbapenemase detection at the time of the case presented here. Consequently, detection of OXA-23 carbapenemase was challenging, since identification of the *bla*_oxa-23_ gene requires specific testing and critical observation of the data from the most common assays (i.e., immunochromatographic and molecular assays for the five main carbapenemases, Hodge test) that may give false-negative results. Prevention of transmission of carbapenemase-resistant *A. baumannii* in hospitals and other health care facilities also involves timely laboratory reporting [22]; hence, the correct identification of OXA- 23 is mandatory to implement the right infection prevention and control measures.

The mucoid *A. baumannii* patient strain seems to have rapidly evolved in order to adapt to the antibiotic regimen administered. The second sample strains will be further genotypically characterized to compare to the first isolates, and to assess the mechanisms responsible for the new resistances and whether subpopulations of hypermutant *A. baumannii* are present. Likewise, viruloma and resistoma data from WGS will be the subject of further studies focused on comparing phenotypic and genomic data.

## Figures and Tables

**Table 1:** Antimicrobial agent susceptibilities of the two *A. baumannii* isolates according to EUCAST interpretative criteria.

	Antimicrobial MIC (µg/mL) and Interpretation for:			
Antimicrobial Agent	*A. baumannii* HM		*A. baumannii* LM	
	MIC	INT	MIC	INT
Ceftazidime/avibactam	>16	-	>16	-
Ceftolozane/tazobactam	32	-	32	-
Ciprofloxacin	≥4	R	≥4	R
Colistin	1	S	0.5	S
Imipenem	≥16	R	≥16	R
Meropenem	≥16	R	≥16	R
Piperacillin/tazobactam	≥32	IE	≥32	IE
Cefotaxime	≥8	-	≥8	-
Ceftazidime	≥16	-	≥16	-
Trimethopim/sulfametoxazole	≥320	R	≥320	R
Cefiderocol	1	IE	0.5	IE
Tigecycline	1	IE	1	IE
Amikacin ^a^	≥32	NA	≥32	NA
Gentamicin ^a^	≥8	NA	≥8	NA
Tobramycin ^a^	≥8	NA	≥8	NA

MIC, minimum inhibitory concentration; HM, hypermucoid; LM, low mucoid; INT, clinical interpretation; R, resistant; S, susceptible; IE, insufficient evidence that the organism is a good target for therapy with the agent; -, no breakpoints available; NA, not available; ^a^ aminoglycoside breakpoints for pulmonary infections are not defined by EUCAST (version 13.0).

## Data Availability

Not applicable.
